# Supplementary material for: Experimental realization of entanglement in multiple degrees of freedom between two quantum memories
Source: Nat Commun. 2016 Nov 14;7:13514. doi: 10.1038/ncomms13514 (PMC5114578; doi:10.1038/ncomms13514)
Supplement: Supplementary Information — Supplementary Figures 1-4, Supplementary Table 1, Supplementary Notes 1-5 and Supplementary References [file ncomms13514-s1.pdf]

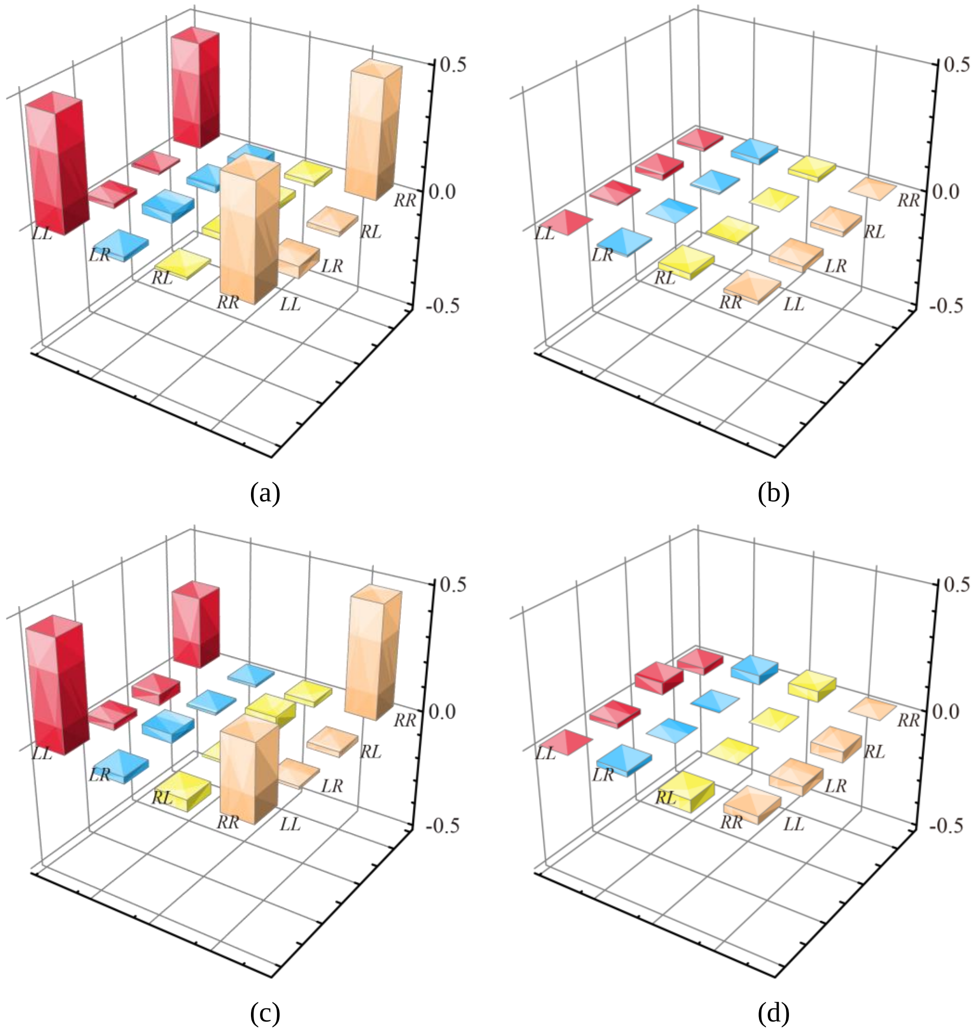

Supplementary Figure 1: Density matrices for 2D OAM entanglement. Real (a, c) and imaginary (b, d) part of the reconstructed density matrix of the 2D OAM entanglement before (a, b) and after (c, d) storage, respectively. All data are raw without any noise correction. The fidelity of entangled state compared with ideal state is 90.7±1.8%, and fidelity of entangled state after storage compared with entangled state before storage is 82±3.0%.

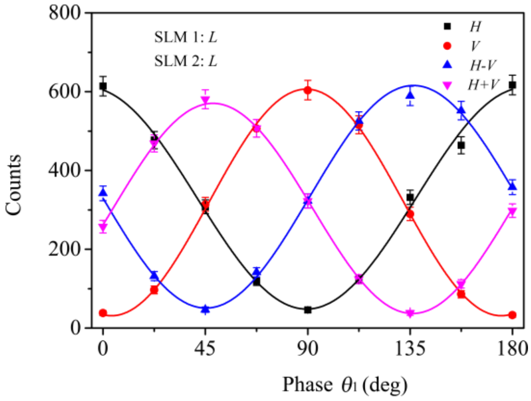
SLM 1: L
SLM 2: L
H
V
H-V
H+V
Counts
Phase θ1 (deg)
0
200
400
600
800
0
45
90
135
180

(a)

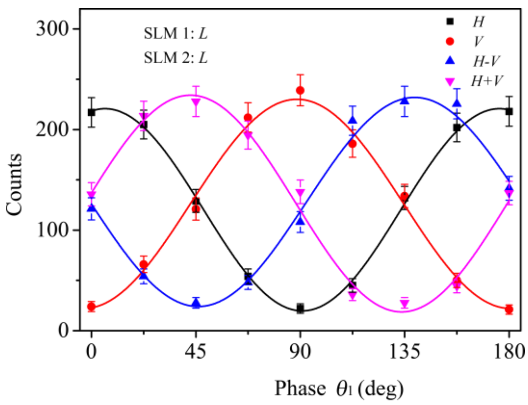
SLM 1: L
SLM 2: L
H
V
H-V
H+V
Counts
Phase θ1 (deg)
0
100
200
300
0
45
90
135
180

(b)

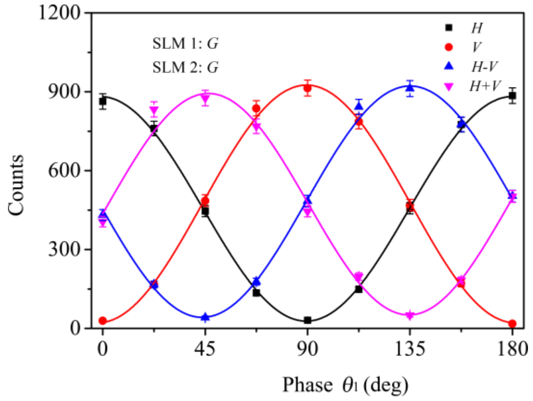
SLM 1: G
SLM 2: G
H
V
H-V
H+V
Counts
Phase θ1 (deg)
0
300
600
900
1200
0
45
90
135
180

(c)

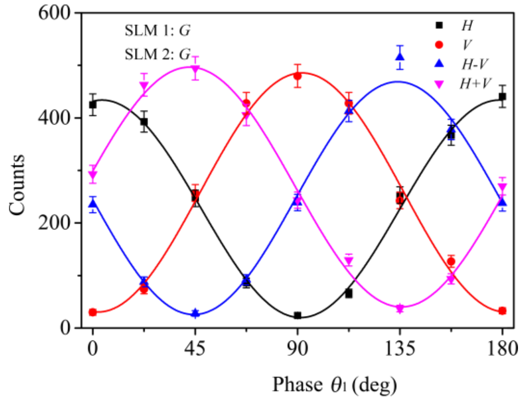
SLM 1: G
SLM 2: G
H
V
H-V
H+V
Counts
Phase θ1 (deg)
0
200
400
600
0
45
90
135
180

(d)

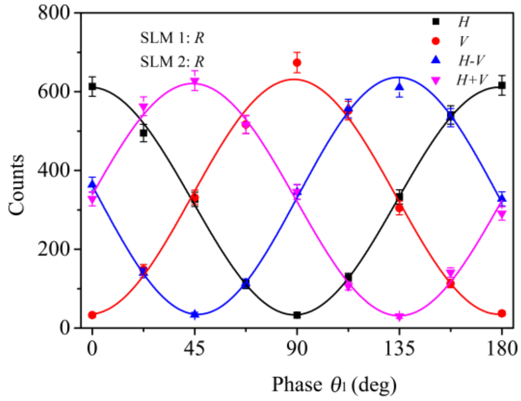
SLM 1: R
SLM 2: R
H
V
H-V
H+V
Counts
Phase θ1 (deg)
0
200
400
600
800
0
45
90
135
180

(e)

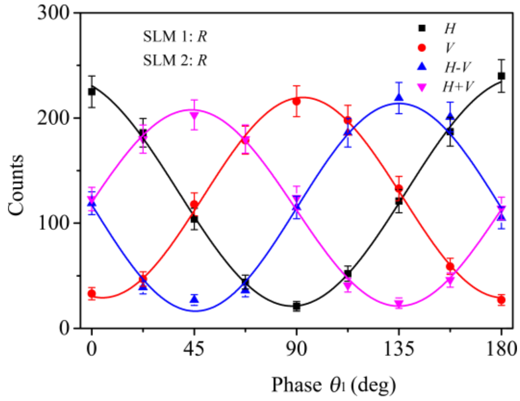
SLM 1: R
SLM 2: R
H
V
H-V
H+V
Counts
Phase θ1 (deg)
0
100
200
300
0
45
90
135
180

(f)

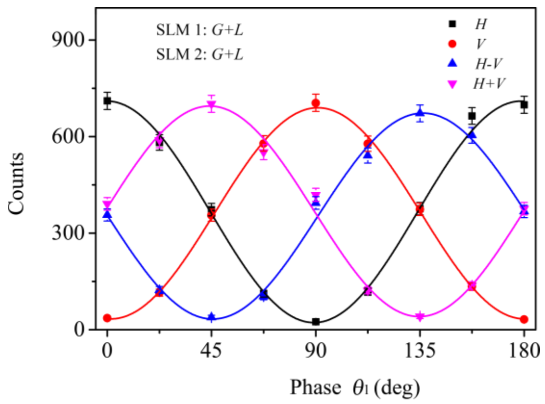
SLM 1: G+L
SLM 2: G+L
H
V
H-V
H+V
Counts
Phase θ1 (deg)
0
300
600
900
0
45
90
135
180

(g)

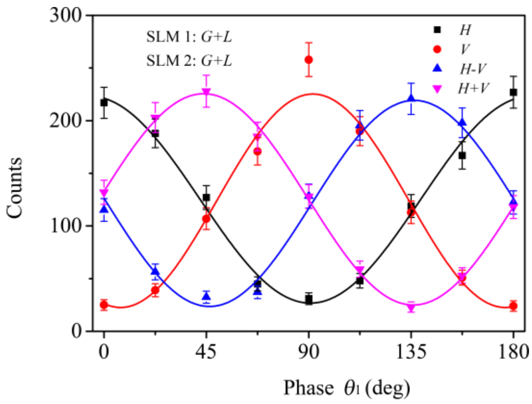
SLM 1: G+L
SLM 2: G+L
H
V
H-V
H+V
Counts
Phase θ1 (deg)
0
100
200
300
0
45
90
135
180

(h)

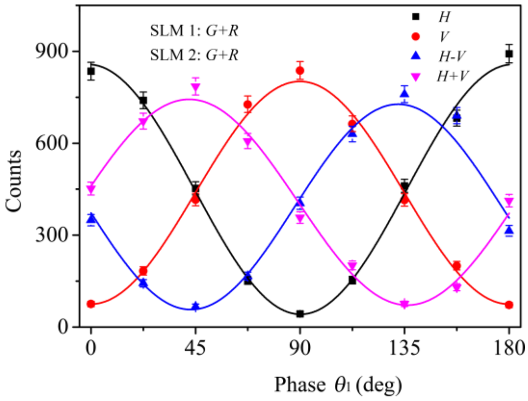
SLM 1: G+R
SLM 2: G+R
H
V
H-V
H+V
Counts
Phase θ1 (deg)

(i)

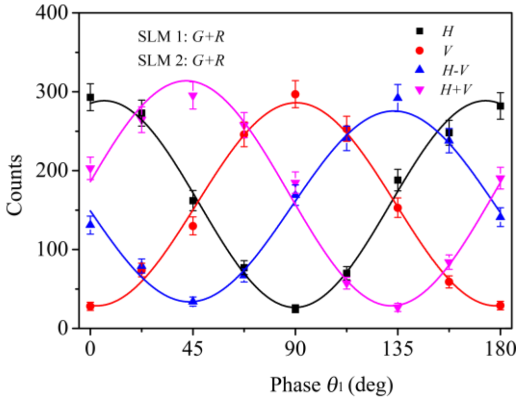
SLM 1: G+R
SLM 2: G+R
H
V
H-V
H+V
Counts
Phase θ1 (deg)

(j)

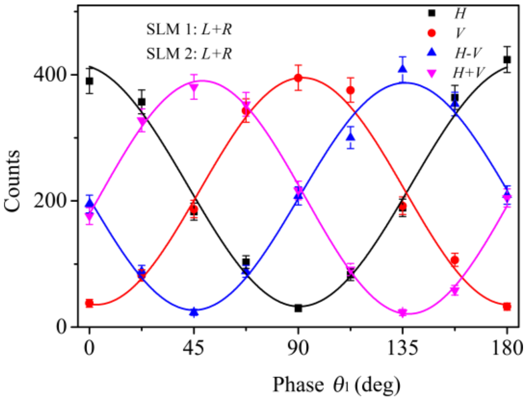
SLM 1: L+R
SLM 2: L+R
H
V
H-V
H+V
Counts
Phase θ1 (deg)

(k)

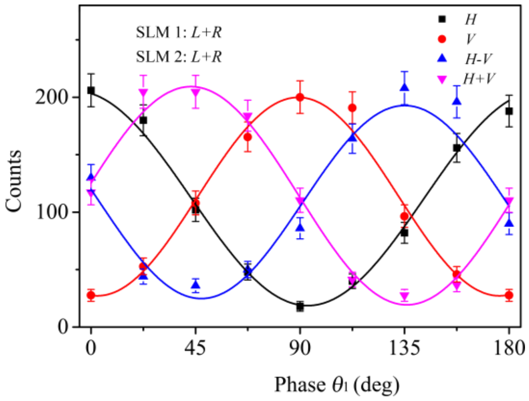
SLM 1: L+R
SLM 2: L+R
H
V
H-V
H+V
Counts
Phase θ1 (deg)

(l)

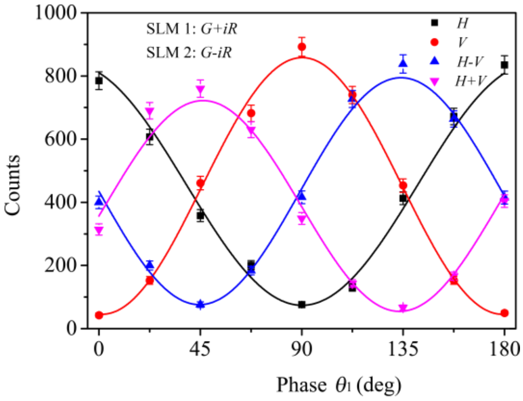
SLM 1: G+iR
SLM 2: G-iR
H
V
H-V
H+V
Counts
Phase θ1 (deg)

(m)

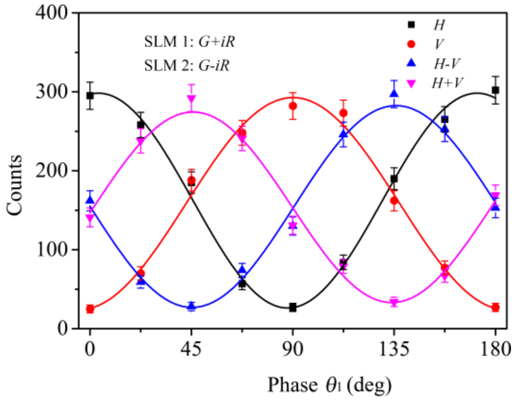
SLM 1: G+iR
SLM 2: G-iR
H
V
H-V
H+V
Counts
Phase θ1 (deg)

(n)

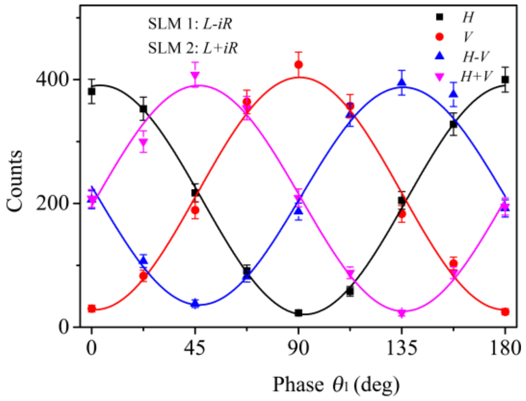
SLM 1: L-iR
SLM 2: L+iR
H
V
H-V
H+V
Counts
Phase θ1 (deg)

(o)

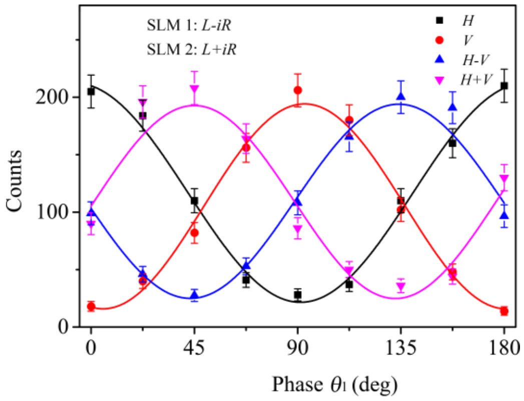
SLM 1: L-iR
SLM 2: L+iR
H
V
H-V
H+V
Counts
Phase θ1 (deg)

(p)

Supplementary Figure 2: Interference curves for polarization entanglement in OAM bases. Interference curves before (a, c, e, g, i, k, m, o) and after (b, d, f, h, j, l, n, p) storage for polarization entanglement when SLMs are projected in the bases of OAM as illustrated. According to the interference curves, we calculate the average visibility in OAM bases accordingly. Before storage, the average visibility is 92.6%, 96.0%, 94.3%, 94.9%, 91.8%, 92.9%, 91%, 93.1% for interference curves in Supplementary Figure 2 (a, c, e, g, i, k, m, o), and after storage, the average visibility is 89.1%, 93.5%, 88%, 87.4%, 90.1%, 86.4%, 89.7%, 86.7% for interference curves in Supplementary Figure 2 (b, d, f, h, j, l, n, p). Each of them is larger than the threshold of 70.7%, which proves the polarization entanglement in the OAM bases. Error bars are estimated from Poisson statistics and represents ±s.d.

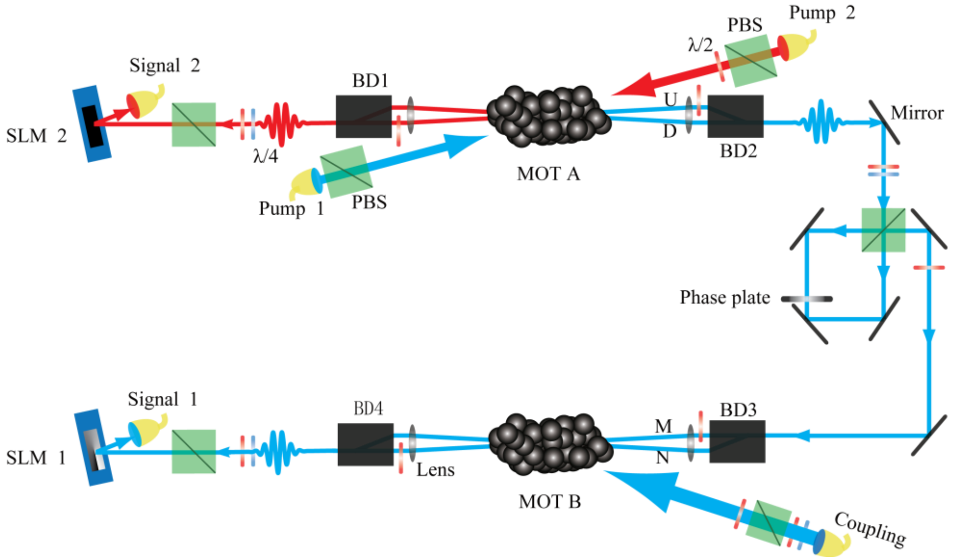

Supplementary Figure 3: Setup of hybrid entanglement. Compared with the setup in Figure 1 for hyperentanglement, there are a little changes for setup of hybrid entanglement including inserting a Sagnac interferometer and adjusting BD2. Here the adjustment of BD2 is for generating another different Bell state as an example to proving the ability of generation of any of the four Bell states.

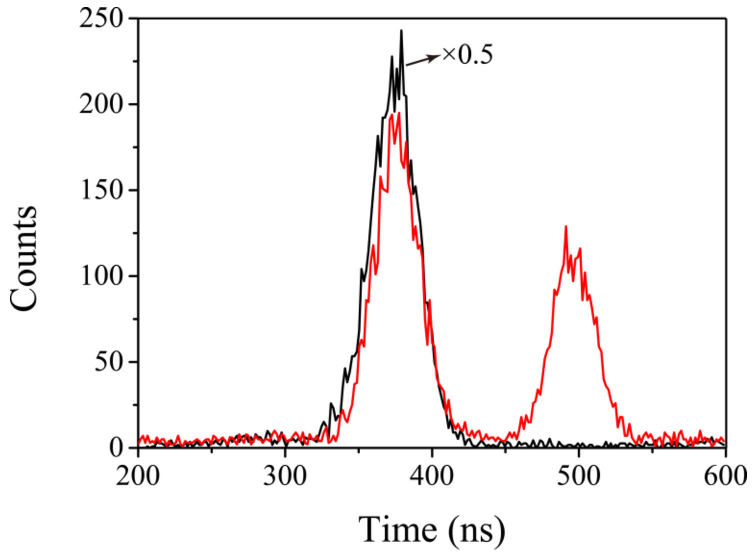

Supplementary Figure 4: The coincidence of Signal 1 and Signal 2 after storage. The black pulse is coincidence (×0.5) only with 200-ns storage in MOT A, the second red pulse is coincidence with 200-ns storage in MOT A and 100-ns storage in MOT B.

SLM 1	SLM 2	Bell value (Before storage)	Bell value (After storage)
|*L*>	|*L*>	2.50±0.05	2.45±0.08
|*G*>	|*G*>	2.60±0.03	2.51±0.05
|*R*>	|*R*>	2.48±0.04	2.44±0.08
|*G*>+|*L*>	|*G*>+|*L*>	2.63±0.04	2.35±0.08
|*G*>+|*R*>	|*G*>+|*R*>	2.42±0.04	2.25±0.07
|*L*>+|*R*>	|*L*>+|*R*>	2.40±0.06	2.39±0.08
|*G*>+*i*|*R*>	|*G*>-*i*|*R*>	2.43±0.04	2.25±0.08
|*L*>-*i*|*R*>	|*L*>+*i*|*R*>	2.38±0.05	2.36±0.07

Supplementary Table 1: The Bell values for polarization entanglement in OAM bases. We verified the violation of Clauser-Horne-Shimony-Holt Bell inequality of polarization entanglement when SLMs are projected in the bases of OAM, the corresponding Bell values are listed above. From the table we can see that, the polarization entanglement exist in the bases of OAM spaces, thus entanglement in polarization DOF and OAM DOF are independent. The

standard deviation of Bell values is estimated from Poisson statistics and using Monte Carlo simulations.

Supplementary Note 1: Independence of entanglement in polarization DOF and OAM DOF

The hyperentanglement consists of two-dimensional (2D) entanglement of spin wave and photonic polarization, three-dimensional (3D) OAM entanglement of spin wave and single photon. Considering completeness for hyperentanglement, we check the various 2D polarization entanglement in the OAM bases to verify the independence between entanglement in different DOFs. Firstly, we construct the density matrix of 2D OAM entanglement before and after storage of Signal-1 photon in horizontal polarization as depicted in Supplementary Figure 1. The related entangled states are listed below. $\left|\psi_{1(\text{OAM-2D})}\right\rangle$ is for the entanglement of Signal 1 and atomic spin wave in MOT A, $\left|\psi_{2(\text{OAM-2D})}\right\rangle$ represents entanglement between two ensembles, and $\left|\psi'_{2(\text{OAM-2D})}\right\rangle$ represents the retrieved photon-photon state after storage.

$$\begin{aligned}\left|\psi_{1(\text{OAM-2D})}\right\rangle &= \left|R_{\text{A}}\right\rangle\left|L_{\text{S1}}\right\rangle + \left|L_{\text{A}}\right\rangle\left|R_{\text{S1}}\right\rangle \\ \left|\psi_{2(\text{OAM-2D})}\right\rangle &= \left|R_{\text{A}}\right\rangle\left|L_{\text{B}}\right\rangle + \left|L_{\text{A}}\right\rangle\left|R_{\text{B}}\right\rangle \\ \left|\psi'_{2(\text{OAM-2D})}\right\rangle &= \left|L_{\text{S2}}\right\rangle\left|L_{\text{S1}}\right\rangle + \left|R_{\text{S2}}\right\rangle\left|R_{\text{S1}}\right\rangle\end{aligned} \tag{1}$$

Next, we explored the Clauser-Horne-Shimony-Holt inequality and plot the interference curves in polarization DOF before and after storage of Signal-1 photon when projecting the SLM 1 and SLM 2 into different bases of OAM space as shown in Supplementary Figure 2. The violation of Clauser-Horne-Shimony-Holt Bell inequality is also verified as the Bell values of polarization entanglement in the OAM bases in shown in Supplementary Table 1.

Supplementary Note 2: Hybrid entanglement

Supplementary Figure 3 is detailed setup for storage of hybrid entanglement. The generated entanglement between Signal 1 and spin wave through SRS process is represented below,

$$\left|\psi_1\right\rangle = \left|D_{\text{A}}\right\rangle\left|V_{\text{S1}}\right\rangle + \left|U_{\text{A}}\right\rangle\left|H_{\text{S1}}\right\rangle \tag{2}$$

where $|D_A>$ and $|U_A>$ refer to the spin wave related to the path *U* and *D* in MOT A accordingly, $|H_{S1}>$and $|V_{S1}>$ represent the generated horizontal and vertical polarization of Signal-1 photon respectively. During this process, the power of SLM 1 is shut down. And the vertical path *M* in MOT B is blocked.

By converting the atom-photon entangled state into photon-photon entangled state and implementing quantum tomography on two-photon polarization entanglement, we measure the coincidence rate based on different basis chosen from $|H>$, $|V>$, $(|H>-i|V>)/2^{\wedge 1/2}$ and $(H>+|V>)/2^{\wedge 1/2}$. The fidelity of density matrix is calculated as 95.3% by comparing with a ideal maximum entangled state. The visibilities on the bases of three mutually unbiased bases of diagonal/anti-diagonal, left-circular/right-circular, and horizontal/vertical bases are 94.2%, 92.3% and 96.4% respectively.

In order to prepare the hybrid entangled state we wanted, the polarization information of signal 1 photons is transferred from polarization DOF into OAM DOF. We input the Signal 1 photons into a special designed Sagnac interferometer, which is inserted with a vortex phase plate with a topological charge of ±1 for oppsite incidenting direction. The entangled state evolves as,

$$\left|\psi'\right\rangle = (\left|D_{\mathrm{A}}\right\rangle\left|V_{\mathrm{S1}}\right\rangle\left|L_{\mathrm{S1}}\right\rangle + e^{i\phi}\left|U_{\mathrm{A}}\right\rangle\left|H_{\mathrm{S1}}\right\rangle\left|R_{\mathrm{S1}}\right\rangle)/\sqrt{2} \tag{3}$$

where, the states $|L>$ and $|R>$ are the OAM states with topological charge of ±1. After Signal 1 photons passing through a half-wave plate with 22.5 °of the optical axis with respect to the vertical axis, the vertical and horizontal states are changed to be diagonal and anti-diagonal polarized state, the output state filtered by a polarization beam splitter can be denoted as,

$$\left|\psi_1\right\rangle = (\left|D_{\mathrm{A}}\right\rangle\left|L_{\mathrm{S1}}\right\rangle + \left|U_{\mathrm{A}}\right\rangle\left|R_{\mathrm{S1}}\right\rangle)/\sqrt{2} \tag{4}$$

Thus the preparation of hybrid entanglement is achieved.

Supplementary Note 3: Fidelity computation

We use the formular $F_1 = Tr(\sqrt{\sqrt{\rho_1}\rho_0\sqrt{\rho_1}})^2$ to calculate fidelity comparing density matrix ρ_1 with ρ_0 .

Supplementary Note 4: Experimental details including coupling efficiencies and counts rate

The coupling efficiency (for both paths *M* and *N*) of Signal 1 from space to fibre is 75%. Signal-1 photons are filtered using three homemade cavities (with temperature control) with 45% transmittance and 70 dB isolation. Signal-2 photons are filtered using two homemade cavities with 65% transmittance and 40 dB isolation. The reflectivity of both SLMs is ~75%. The efficiency of every single photon detector is ~50%.

During our experiment, the dark-count rate is ~180/s for both detectors while the photon counts rate (including dark-count rate) is ~800/s. The overall rate of successful events is 2.5/s accounting for ~500 coincidence counts (in the bases of H-H or L-L) in total 200 s. So, on each 500-ns run, the probability (corrected for loss) of generating entanglement between two atomic ensembles is estimated as $2.5/(2800\times100\times75\%\times65\%\times45\%\times50\%\times50\%\times75\%\times75\%)\sim2.9\times10^{-4}$, in which 2800×100 is the trial number per second, the first 75% is coupling efficiency for Signal 1 from space to fibre, $65\%\times45\%$ is for cavity transmission efficiency, the $50\%\times50\%$ is the total detecting efficiencies of two detectors, $75\%\times75\%$ is the total reflectivity of two SLMs.

Assuming an entangled pair is produced, the probability of detecting a successful event is $1\times75\%\times65\%\times45\%\times50\%\times50\%\times75\%\times75\%=3.1\times10^{-2}$.

Supplementary Note 5: Memory performance

Because our magnetic field for trapping can't be shut down completely within 1.4 ms due to big value of inductance, our memory time is rather limited to 1.4 μs as discussed before in previous work[1], the 100-ns memory efficiency in MOT B is ~25% for photon carrying no OAM information as depicted in Supplementary Figure 4 and is ~20% for photon carrying OAM with topological value $l=\pm1$. In general, memory time can be improved by compensating the magnetic field or by using magnetic field-insensitive states and reducing atomic motion by using optical lattice, a millisecond and even hundred millisecond storage time could be achieved[2-5]. In addition, the dynamic decoupling method can also be used to improve the storage time[6]. Memory efficiency can be increased by using waveform modulation [7].

Supplementary Reference

1. Ding, D.-S. *et al.* Quantum Storage of Orbital Angular Momentum Entanglement in an Atomic Ensemble. *Phys. Rev. Lett.* **114**, 050502 (2015).

2. Zhao, B. *et al*. A millisecond quantum memory for scalable quantum networks. *Nat. Phys.* **5**, 95-99 (2009).

3. Xu, Z.-X. *et al*. Long lifetime and high-fidelity quantum memory of photonic polarization qubit by lifting Zeeman degeneracy. *Phys. Rev. Lett.* **111**, 240503 (2013).

4. Radnaev, A. G. *et al*. A quantum memory with telecom-wavelength conversion. *Nat. Phys.* **6**, 894-899 (2010).

5. Yang, S.-J., Wang, X.-J., Bao, X.-H. & Pan, J.-W. An efficient quantum light–matter interface with sub-second lifetime. *Nat. Photon.* **10**, 381-384 (2016).

6. Heinze, G. *et al*. Stopped light and image storage by electromagnetically induced transparency up to the regime of one minute. *Phys. Rev. Lett.* **111**, 033601 (2013).

7. Chen, Y.-H. *et al*. Coherent Optical Memory with High Storage Efficiency and Large Fractional Delay. *Phys. Rev. Lett.* **110**, 083601 (2013).
